# Long noncoding RNAs and mRNAs profiling in ovary during laying and broodiness in Taihe Black-Bone Silky Fowls (*Gallus gallus Domesticus Brisson*)

**DOI:** 10.1186/s12864-024-10281-7

**Published:** 2024-04-10

**Authors:** Yuting Tan, Yunyan Huang, Chunhui Xu, Xuan Huang, Shibao Li, Zhaozheng Yin

**Affiliations:** Zijingang Campus, Animal Science College, Zhejiang University, Hangzhou, 310058 China

**Keywords:** Broodiness, Egg production, lncRNA, Transcriptomes, Ovarian follicle development

## Abstract

**Background:**

Broodiness significantly impacts poultry egg production, particularly notable in specific breeds such as the black-boned Silky, characterized by pronounced broodiness. An understanding of the alterations in ovarian signaling is essential for elucidating the mechanisms that influence broodiness. However, comparative research on the characteristics of long non-coding RNAs (lncRNAs) in the ovaries of broody chickens (BC) and high egg-laying chickens (GC) remains scant. In this investigation, we employed RNA sequencing to assess the ovarian transcriptomes, which include both lncRNAs and mRNAs, in eight Taihe Black-Bone Silky Fowls (TBsf), categorized into broody and high egg-laying groups. This study aims to provide a clearer understanding of the genetic underpinnings associated with broodiness and egg production.

**Results:**

We have identified a total of 16,444 mRNAs and 18,756 lncRNAs, of which 349 mRNAs and 651 lncRNAs exhibited significantly different expression (DE) between the BC and GC groups. Furthermore, we have identified the cis-regulated and trans-regulated target genes of differentially abundant lncRNA transcripts and have constructed an lncRNA-mRNA trans-regulated interaction network linked to ovarian follicle development. Gene Ontology (GO) and Kyoto Encyclopedia of Genes and Genomes (KEGG) annotation analyses have revealed that DE mRNAs and the target genes of DE lncRNAs are associated with pathways including neuroactive ligand-receptor interaction, CCR6 chemokine receptor binding, G-protein coupled receptor binding, cytokine-cytokine receptor interaction, and ECM-receptor interaction.

**Conclusion:**

Our research presents a comprehensive compilation of lncRNAs and mRNAs linked to ovarian development. Additionally, it establishes a predictive interaction network involving differentially abundant lncRNAs and differentially expressed genes (DEGs) within TBsf. This significantly contributes to our understanding of the intricate interactions between lncRNAs and genes governing brooding behavior.

**Supplementary Information:**

The online version contains supplementary material available at 10.1186/s12864-024-10281-7.

## Background

Broodiness is a common behavior observed in domestic fowl, which can hinder egg production and impact the poultry industry [1]. This behavior is prevalent across various breeds of domestic fowl, with black chickens exhibiting particularly strong broodiness tendencies. Typically, broodiness occurs after approximately every 15 eggs are laid, leading to a cessation in egg laying [2]. Broodiness is often characterized by elevated body temperature, decreased feed and water intake, frequent occupancy of nesting areas, the act of turning and retrieving eggs, aggressive or defensive behaviors, distinctive clucking sounds, and a halt in egg production [3]. Moreover, broodiness is influenced by genetic factors as well as aspects such as nutrition and hormonal regulation. Genetic predisposition is recognized as the primary cause of broodiness in fowl.

The broodiness behavior of poultry is largely regulated by the hypothalamic-pituitary-gonad (HPG) axis, with the dynamic changes in ovarian signals playing a significant role in determining the activities of the hypothalamic-pituitary unit [4]. The ovary serves as a key reproductive organ in vertebrates, responsible for the production and daily release of oocytes. It comprises follicles at various developmental stages, each consisting of a central oocyte surrounded by endocrine cells [5]. The domestic fowl provides a unique model for studying follicular development. Unlike mammals, the hen's single left ovary contains follicles of diverse sizes and developmental stages. From resting primordial follicles to pre-hierarchical growing follicles, pre-ovulatory follicles, and post-ovulation follicles, various stages of follicular development can coexist within an ovary exhibiting reproductive activity. Prior to ovulation, follicle size can increase from 6–8 mm to 40 mm within a span of 5–9 days [6]. Examining the ovary provides a starting point for exploring the molecular mechanisms underlying hen broodiness, thus offering a theoretical basis for enhancing egg production.

The development of the ovary and follicles plays a crucial role in determining the laying performance of chickens. Biological processes governing ovarian development and ovulation are regulated by a plethora of dynamically and stage-specifically expressed key genes [7]. Therefore, investigating the transcriptional expression patterns of chicken ovarian tissue during broodiness can offer valuable insights into screening and identifying key genes involved in regulating chicken ovarian development. With the advancement of high-throughput sequencing technologies, transcriptomic analyses have been utilized to identify candidate genes, pathways, and molecular mechanisms underlying broodiness.

Transcriptome sequencing is an effective approach for exploring the significant roles of lncRNAs, which are RNA transcripts longer than 200 nucleotides and do not encode proteins; they are broadly classified as lncRNAs [8, 9]. LncRNAs may participate in various cellular processes such as mRNA splicing and maturation, mRNA transport or localization, and mRNA stabilization [10].

We focus on RNA transcripts longer than 200 nucleotides in ovaries, which have described noncoding functions. LncRNAs are widely recognized as playing crucial roles in reproductive processes, including sexual maturation [11], spermatogenesis [12], and premature ovarian insufficiency [13]. Wang et al. identified 1182 mRNAs and 168 lncRNAs that differed in granulosa cells of small yellow follicles from Jinghai Yellow chickens in red light and white light groups. Many of these genes are involved in follicular development, including pathways related to steroid hormone synthesis, oocyte meiosis, and the PI3K-Akt signaling pathway [14]. Meanwhile, Wu et al. constructed lncRNA-gene interaction networks involving 34 differentially abundant lncRNAs and 263 DEGs, leading to an enhanced understanding of lncRNA and gene interactions regulating broodiness [15]. Taken together, the integrated analysis of lncRNAs and mRNAs may provide novel insights into the mechanisms regulating broodiness.

The TBsf originates from Wangbantu village, Taihe county, Jiangxi province. It is characterized by features such as a tassel head, tufted crown, green ear, beard, silk hair, black skin, black meat, black bone, hairy foot, and five claws. This breed holds significant value in terms of its edibility, medicinal properties, and ornamental appeal, making it a valuable genetic resource and traditional Chinese medicine [16, 17]. TBsf has long been esteemed as a health-promoting food with various therapeutic and pharmacological effects, including antioxidative and anti-fatigue properties. Broodiness, a natural instinct for nurturing offspring, is particularly pronounced in TBsf. Under natural conditions, TBsf typically exhibits broodiness once for every 10–12 eggs laid, with each episode lasting more than 15 days. However, this behavior poses challenges for the economic development of the TBsf industry. To date, Liao et al. have investigated muscle metabolites in TBsf influenced by breed and feed traits at the metabolome level [18]. Additionally, Xiang et al. have elucidated the differentially expressed genes and pathways impacting egg production performance at the transcriptome level. Despite these advances, the specific roles of lncRNAs in the reproductive processes of TBsf remain largely undefined. There is a compelling need for further investigations to elucidate the contributions of lncRNAs and mRNAs to ovarian development.

In our recent study, we analyzed two distinct phases: the broodiness phase and the peak egg-laying phase in hens to assess the influence of broodiness on egg production rates. We utilized RNA sequencing to examine the variances in ovarian lncRNAs and mRNAs across these phases. This comparison allowed us to identify lncRNAs and mRNAs that were differentially abundant. We then conducted a bioinformatic analysis on the selected lncRNAs to predict potential cis- and trans-target genes, which facilitated the construction of related lncRNA gene networks. Further, we performed functional and pathway enrichment analyses on pivotal genes using GO and the KEGG tools. Our findings hold promise for unraveling the molecular underpinnings of broodiness in black chickens and could contribute to enhancing their egg-laying performance.

## Results

### RNA sequencing and mapping

Eight ovarian tissue libraries were constructed to investigate lncRNAs and mRNAs in laying and nesting TBsf. Following rigorous quality control measures, which involved the removal of reads containing adapter contamination, undetermined bases, and low-quality sequences, each sample yielded approximately 83 to 104 million clean reads from an initial count of 86 to 106 million raw reads. The data exhibited high quality, with Q20 scores ranging from 96.86% to 97.46%, Q30 scores from 91.57% to 92.80%, and GC content between 47.07% and 47.62%. Notably, approximately 94% of the clean reads across all libraries were successfully aligned with the reference genome, with 90% of these reads uniquely mapped (Table S1).

### Transcriptome assembly

**Fig. 1:**
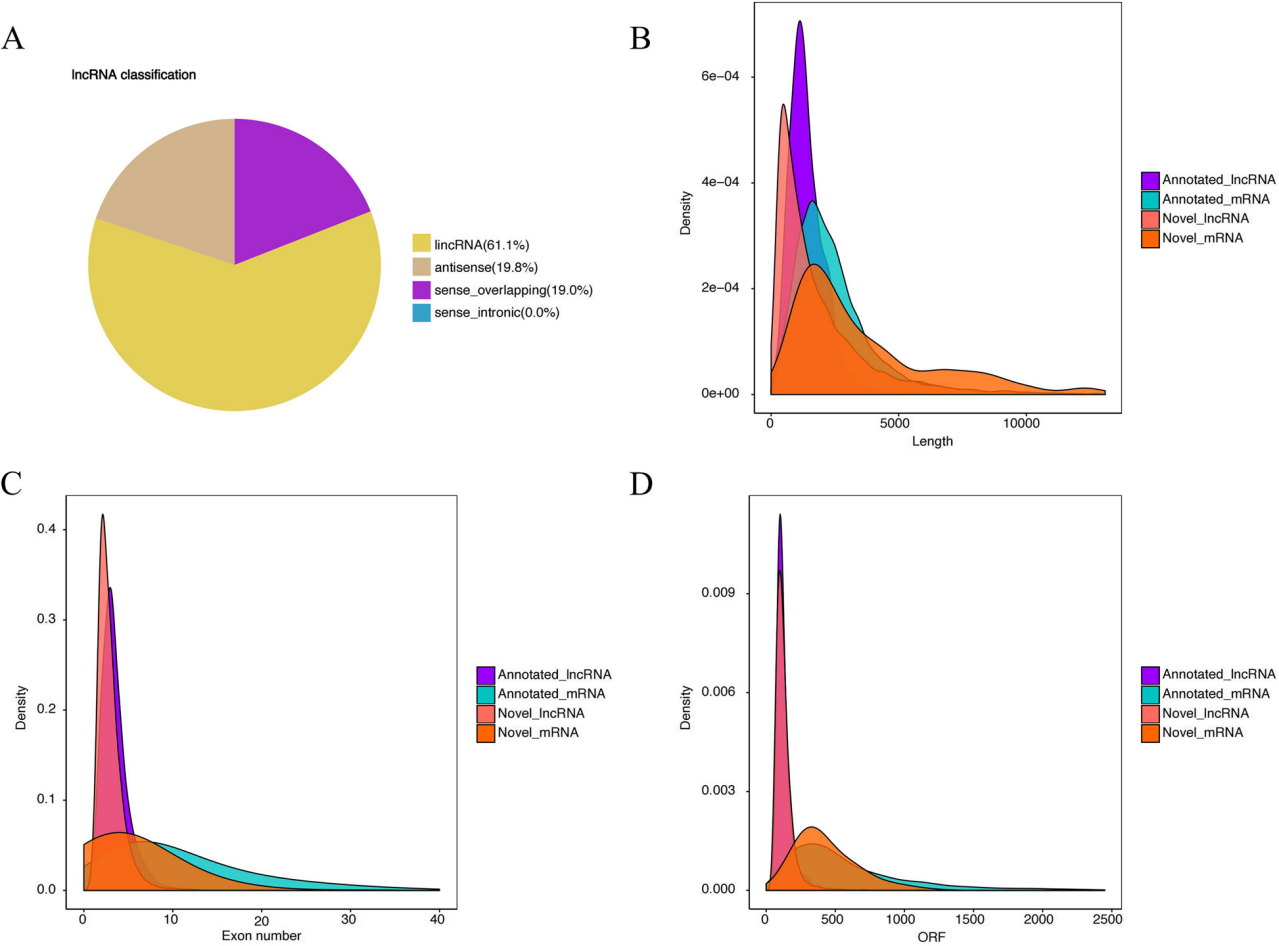
LncRNA classification and genomic features in the ovaries of TBsf. **A** LncRNA classification. **B** The transcript length distribution of lncRNAs and mRNAs. **C** The exon number distribution of lncRNAs and mRNAs. **D** The ORFs length distribution of lncRNAs and mRNAs

In the current research, the assembly of transcripts revealed a total of 21,425 lncRNAs, encompassing 8,870 known and 12,555 novel entities. The protein-coding potential of these transcripts was assessed using computational tools such as CPC2, CNCI, and Pfam. The analysis delineated the genomic distribution of the novel lncRNAs, revealing that 7,677 (61.1%) were intergenic, 2,490 (19.8%) were identified as antisense lncRNAs, and 2,388 (19.0%) as sense-overlapping lncRNAs (Fig. 1A). The investigation also demonstrated that the average length of lncRNA transcripts is 2,049 base pairs, which is significantly shorter than the average length of mRNA transcripts, documented at 5,607 base pairs, underscoring the characteristic brevity of lncRNAs compared to mRNAs (Fig. 1B). Additionally, it was observed that lncRNAs possess fewer exons, averaging 3.05, in contrast to the average of 5.03 exons in mRNAs (Fig. 1C). Moreover, in ovarian tissue, lncRNAs exhibited shorter open reading frames (ORFs) compared to mRNAs (Fig. 1D), further contributing to the understanding of their structural and functional attributes.

### Differentially expressed mRNAs and lncRNAs

**Fig. 2:**
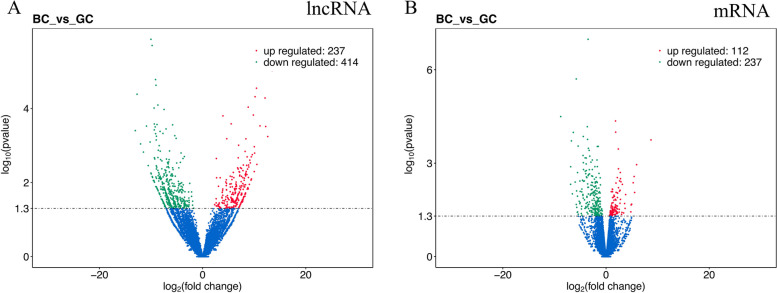
Differential expression of lncRNAs and mRNAs between the BC and GC. Up-regulated genes are shown in red, down-regulated genes are shown in green, and genes with no significant difference in expression are indicated in blue; significance was indicated by a p-value < 0.05. **A** Differential expression of lncRNAs. **B** Differential expression of mRNAs

To discern the DE lncRNAs and mRNAs between the BC and GC groups, edgeR software was employed for analysis. This process elucidated a significant differential expression of 651 lncRNAs and 349 mRNAs between the ovarian tissues of the BC and GC groups (Tables S2 and S3). Among these, 237 lncRNAs and 112 mRNAs exhibited significant upregulation, while 414 lncRNAs and 237 mRNAs demonstrated downregulation in the comparative analysis (Fig. 2A, B).

### Enrichment analysis of differentially expressed mRNAs

**Fig. 3:**
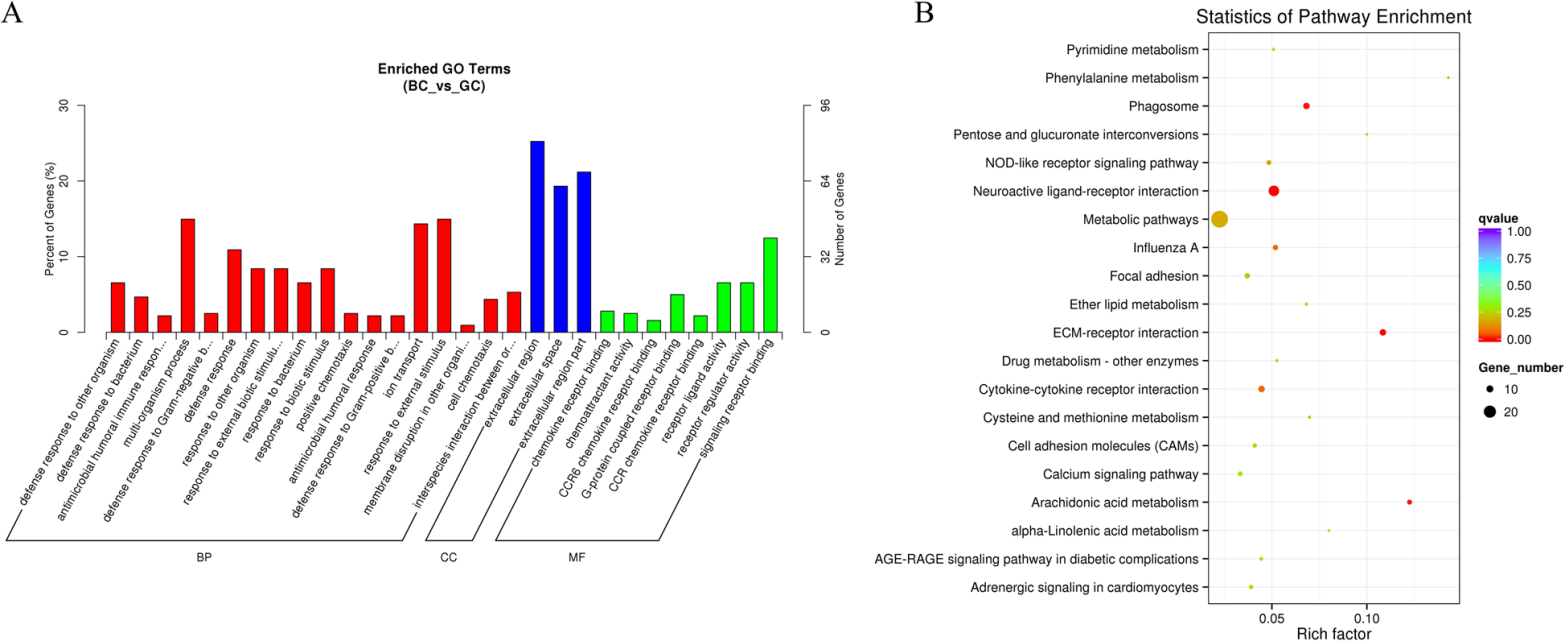
GO and KEGG analysis of differential mRNA expression. **A** Histogram of GO enrichment of DE mRNAs. **B** Scatter plot of KEGG enrichment for DE mRNAs

The GO database serves as an exhaustive resource for annotating gene functions, systematically divided into three principal categories: biological processes (BPs), cellular components (CCs), and molecular functions (MFs). In the analysis comparing BC and GC groups, a total of 321 differentially expressed mRNAs were correlated with 4,850 GO terms bearing functional annotations. These encompassed 3,745 BPs, 392 CCs, and 713 MFs. Notably, among these, 29 GO terms were identified as significantly enriched, adhering to the criterion of a q-value < 0.05 (Table S4). The significantly enriched terms included noteworthy entries such as extracellular region, chemokine receptor binding, multi-organism process, CCR6 chemokine receptor binding, and G-protein coupled receptor binding (Fig. 3A). Furthermore, KEGG pathway analysis elucidated four significantly enriched pathways (q-value < 0.05), namely ECM-receptor interaction, Neuroactive ligand-receptor interaction, Arachidonic acid metabolism, and Phagosome (Fig. 3B), with detailed information catalogued in Table S5.

### Regulatory roles of differentially expressed lncRNAs in ovaries

**Fig. 4:**
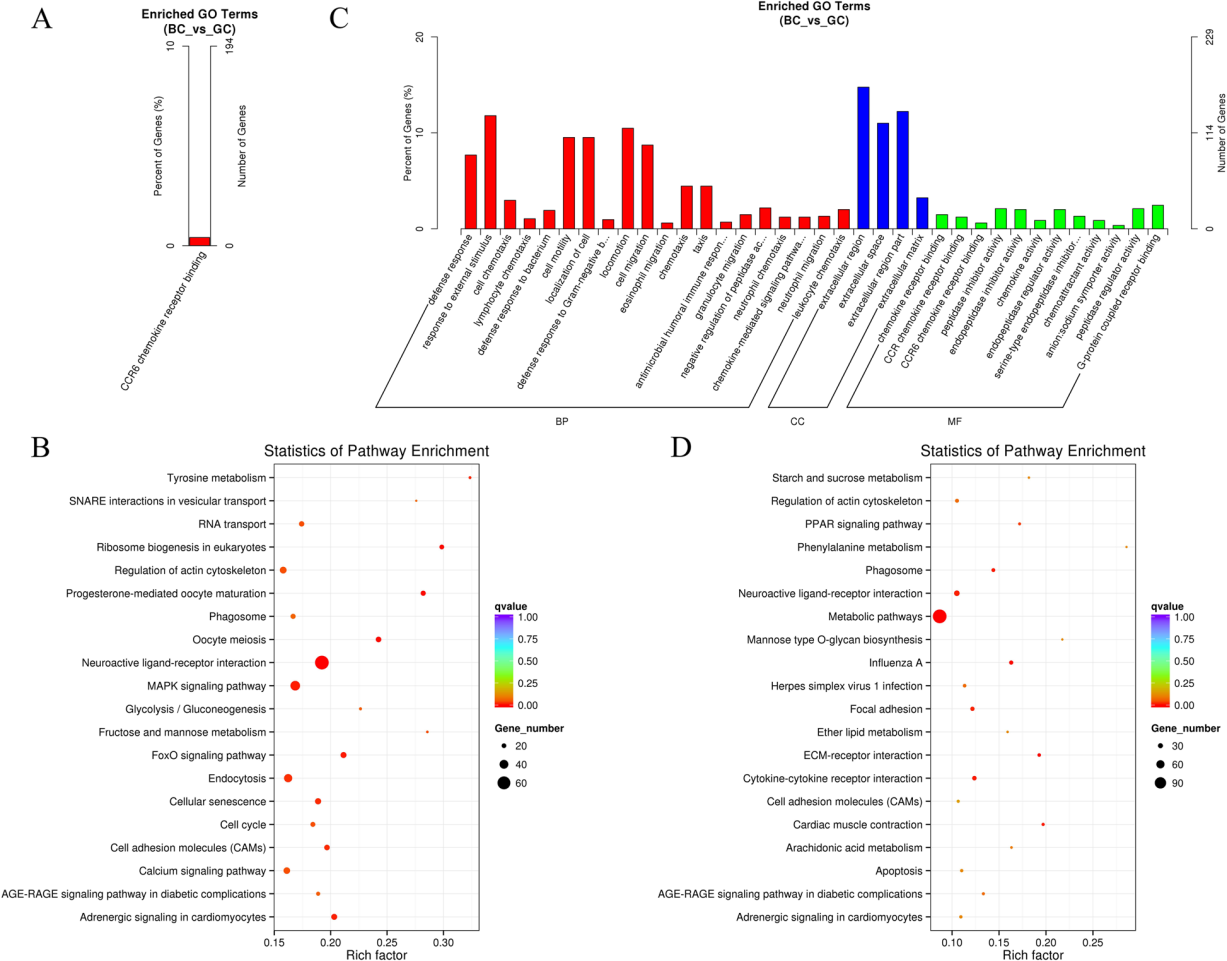
GO and KEGG analysis of differential lncRNAs target gene. **A** Histogram of GO enrichment of target gene of DE lncRNAs in cis-regulatory. **B** Scatter plot of KEGG enrichment of target gene of DE lncRNAs in cis-regulatory. **C** Histogram of GO enrichment of target gene of DE lncRNAs in trans-regulatory. **D** Scatter plot of KEGG enrichment of target gene of DE lncRNAs in trans-regulatory

To elucidate the regulatory roles of lncRNAs, this study predicted potential cis-acting and trans-acting targets among the DE lncRNAs between BC and GC groups. In the context of cis-acting regulation, we identified protein-coding genes situated within a 100 kb range upstream and downstream of the lncRNAs. This approach culminated in the discovery of 2,163 potential cis-regulated target genes (Table S6). Subsequent GO analysis underscored one significantly enriched GO term (q-value < 0.05), revealing an association of the DE lncRNA target genes with functionalities pertinent to CCR6 chemokine receptor binding (Fig. 4A, Table S7). Moreover, pathway analysis highlighted these cis-regulated target genes of lncRNAs as being enriched in 16 KEGG pathways, notably those linked with ovarian follicle development, such as Neuroactive ligand-receptor interaction, Progesterone-mediated oocyte maturation, Ribosome biogenesis in eukaryotes, Oocyte meiosis, and the MAPK signaling pathway (Fig. 4B, Table S8). These observations support the premise that lncRNAs may influence ovarian follicle development through cis-regulatory effects on adjacent protein-coding genes.

In the examination of trans-regulated target genes of DE lncRNAs, this investigation identified 1,302 potential target genes. The lncRNAs co-expressed with these genes manifested significant enrichment in 41 GO terms, comprising 25 biological processes (BP), 4 cellular components (CC), and 12 molecular functions (MF), encapsulating a diverse array of biological processes (q-value < 0.05). Prominent terms included extracellular region, chemokine receptor binding, defense response, response to external stimulus, and cell motility (Fig. 4C, Tables S9 and S10). Furthermore, KEGG pathway analysis was applied to the differentially abundant trans lncRNAs to elucidate pathways augmented by the expression of these genes. The analysis revealed that differentially abundant lncRNAs, co-expressed with protein-coding genes, were significantly enriched in nine KEGG pathways. These pathways include metabolic pathways, ECM-receptor interaction, cytokine-cytokine receptor interaction, neuroactive ligand-receptor interaction, and focal adhesion (Fig. 4D, Table S11). These results infer that lncRNAs may influence ovarian follicle development through trans-regulatory effects on non-adjacent protein-coding genes.

### Target gene prediction of lncRNAs and interaction network construction

**Fig. 5:**
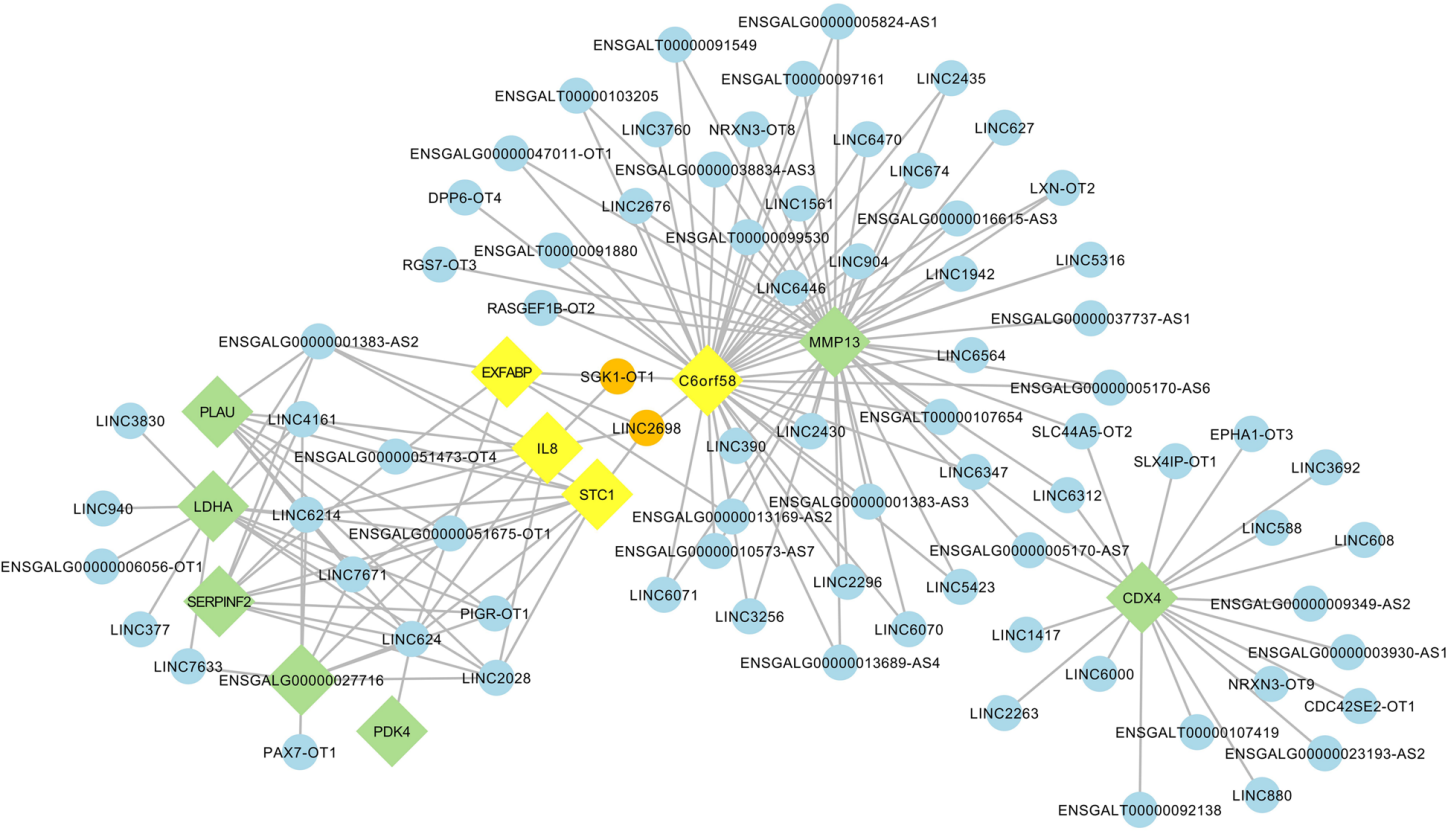
Interacting network of lncRNAs and their trans-regulated target genes associated with ovarian follicle development. The blue circles and green squares represent lncRNAs and potential target genes, respectively, where orange circles and yellow squares denote central nodal lncRNAs and target genes

To elucidate the biological mechanisms mediated by putative lncRNAs in the context of ovarian follicle development, bioinformatics tools were employed to construct a regulatory network encompassing these lncRNAs and their potential target genes. Acknowledging the predominant mode of action of most lncRNAs via trans-regulatory pathways, we developed an lncRNA-mRNA trans-regulatory interaction network specifically associated with ovarian follicle development (Fig. 5). This network diagram delineates the complex regulatory interconnections between the identified lncRNAs and their corresponding trans-regulated target genes. Through detailed analysis of the network structure, we pinpointed two pivotal nodal lncRNAs and four key nodal genes situated at the convergence of two principal regulatory circuits, underscoring their potential significance in the modulation of ovarian follicle development.

### qRT-PCR validation of mRNAs and lncRNAs

**Fig. 6:**
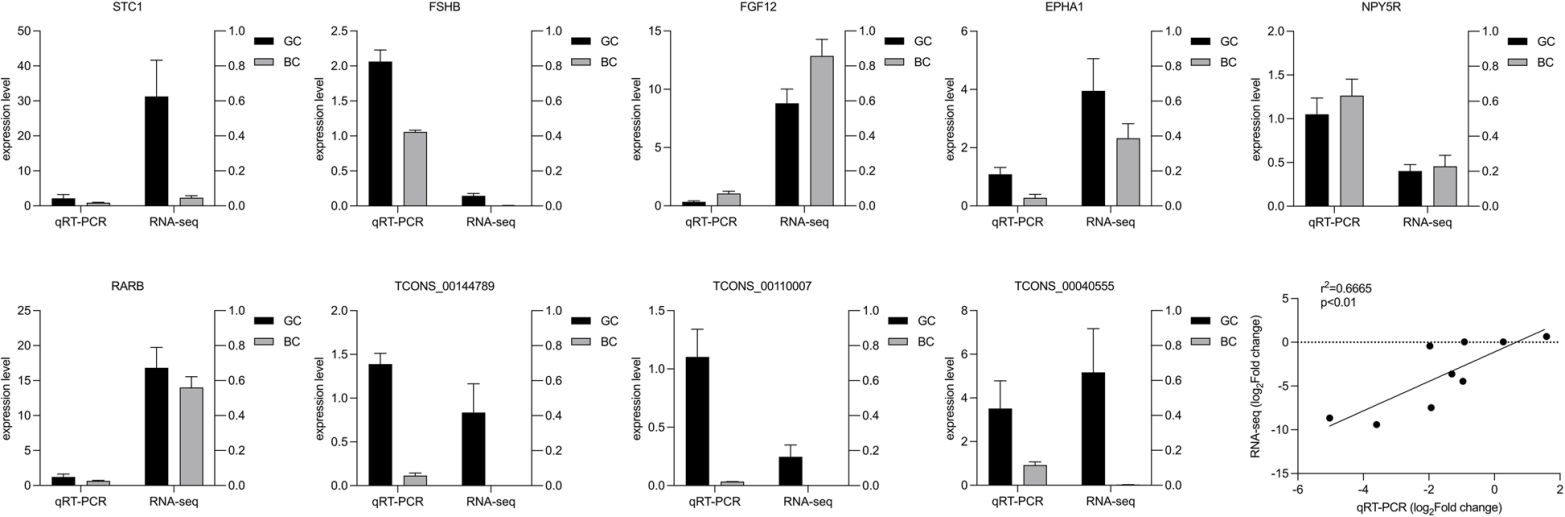
Validation of RNA-seq by qRT-PCR

To corroborate the findings from RNA sequencing, we conducted quantitative real-time PCR (qRT-PCR) analysis on a subset comprising six mRNAs and three lncRNAs. The expression patterns delineated in Fig. 6 demonstrate congruence between the qRT-PCR outcomes and those derived from the RNA-seq data. The analysis yielded a Pearson correlation coefficient of 0.6665 between the datasets pertaining to the six mRNAs and three lncRNAs, substantiating the accuracy and reliability of the RNA-seq results.

## Discussion

Egg production is a critical parameter for assessing the reproductive performance of hens. However, nesting behavior in hens can lead to decreased egg production, presenting a potential area of economic interest. Exploring the behavioral mechanisms underlying chicken nesting is essential for enhancing egg production through genetic and breeding innovations. Nesting behavior is regulated by many critical genes and complex regulatory pathways. Yet, the specific mechanisms governing broodiness in TBsf remain ambiguous. Egg production traits are related to ovarian function and are regulated by the HPG axis [19]. Therefore, ovarian tissue was selected to perform the RNA-seq analysis. At present, many studies have found that lncRNAs and mRNAs play critical roles in the regulation of ovary development. Ovary lncRNAs and mRNAs have been identified in chickens [11], Yili geese [20], Hu sheep [21], Muscovy ducks [15], domestic pigeons [22] and Chinese Tongue Sole [23]. Although the mRNA transcriptome in TBsf ovary has been partially explored in previous studies [24], a systematic evaluation of the lncRNAs and mRNAs across TBsf ovary developmental stages and broodiness stages is lacking. In this study, we constructed eight cDNA libraries from the ovaries of BC and GC groups to evaluate the expression of mRNAs and lncRNAs by Illumina high-throughput sequencing, and we identified critical candidate lncRNAs related to broodiness. Specifically, we identified 349 DE mRNAs and 651 DE lncRNAs between BC and GC groups fowl ovaries. In addition, we found that lncRNAs identified in this study have shorter transcript lengths, fewer exons and shorter ORFs than protein coding transcripts, which indicated that the lncRNAs were of high quality and that the data collected were reliable.

In this study, we identified a fascinating group of lncRNAs and their target genes by integrating the interaction network of differentially expressed lncRNAs and their trans-regulated target genes. This group includes SGK1-OT1 and its target genes IL8, C6orf58 and EXFABP, as well as LINC2698 and its target genes STC1, IL8, C6orf58, and EXFABP. These genes are located at the junctures of two large regulatory loops, garnering our attention for further research.

SGK1-OT1, a long non-coding RNA (lncRNA), functions as a regulatory molecule associated with SGK1, a serum and glucocorticoid-regulated kinase 1 known to play a significant role in various biological processes. This kinase is crucial in responding to dehydration, salt load, and other forms of environmental stress [25]. Although the specific role of SGK1-OT1 in ovarian development remains unclear, the related gene SGK1 has been implicated in the progression of ovarian cancer and the development of resistance to treatment [26, 27]. Overexpression of SGK1 promotes tumor cell proliferation and migration while inhibiting induced apoptosis in cancer, suggesting a potential role for SGK1, and by extension SGK1-OT1, in promoting cell survival and proliferation—traits characteristic of cancer cells. Most research has not directly explored the role of SGK1 in chicken egg production or other reproductive behaviors; however, given the importance of SGK1 in stress response and tumor biology in other mammals [28, 29], it can be speculated that SGK1 may indirectly affect chicken egg-laying behaviors and reproductive performance by influencing cell growth and survival. This hypothesis, however, requires further investigation for a deeper understanding. Studies specifically focusing on how SGK1 affects chicken reproductive performance are limited. More research is needed in this area. Currently, there is no research directly linking LINC2698 to reproduction, but its regulation closely associated with SGK1-OT1 warrants further exploration. The role of lncRNAs in biology is often complex and context-dependent, regulated by numerous factors. Therefore, further studies are necessary to elucidate the specific roles of SGK1-OT1 and LINC2698 in chicken ovarian development.

In this study, SGK1-OT1 and LINC2698 primarily exert their biological functions through the trans-regulation of STC1, IL8, C6orf58, and EXFABP. Stanniocalcin-1 (STC-1) is a widely expressed glycoprotein hormone involved in various physiological and pathological processes, including angiogenesis, mineral homeostasis, cell proliferation, inflammation, and apoptosis. Within female reproductive tissues, STC-1 expression is associated with processes such as ovarian follicle development, embryonic implantation, early pregnancy vascular remodeling, and placental development [30]. STC-1 is recognized as a crucial ovarian regulatory factor, promoting ovarian development in mice. It stimulates granulosa cell proliferation and affects the secretion of E2 (estradiol) and P4 (progesterone) by influencing the expression of key genes involved in steroid hormone synthesis [31].Interleukin-8 (IL-8), also known as CXCL8, is a well-known chemotactic cytokine secreted by various cells. Recent studies have demonstrated the involvement of IL-8 in the establishment of the corpus luteum following ovulation. IL-8 contribute to angiogenesis and IL-8 specifically stimulates progesterone secretion through its actions on luteinized granulosa cells and luteal membrane cells [32]. IL-8 also participates in the regulation of inflammatory responses and immune modulation in ovarian tissues. Aberrant expression of IL-8 may be associated with the occurrence and progression of ovarian diseases, including polycystic ovary syndrome and ovarian tumors [33, 34]. C6orf58 (LEG1) is identified as a human gene encoding a protein known as UPF0762, which is secreted following the cleavage of its signal peptide. Early proteomics studies have identified a homologous protein annotated as C6orf58 in the ovarian fluid of rainbow trout, participating in lipid binding and metabolism, carbohydrate and ion transport, innate immunity, maturation, and reproduction alongside other proteins [35]. Additionally, C6orf58 has been detected in the seminal plasma of ejaculated ram sperm, with enrichment analysis indicating key molecular functions in enzyme regulation, catalytic activity, binding, and receptor activity. The majority of the literature published to date links LEG1 with liver function and development [36–38], without finding definitive evidence of its direct involvement in the ovarian development process. EXFABP, also referred to as Exogenous Fatty Acid-Binding Protein (Ex-FABP), is a unique protein predominantly found in chicken serum, selectively binding and transporting fatty acids within the extracellular fluid and blood. Ex-FABP is expressed during chicken embryo development, notably within hypertrophic chondrocytes, muscle fibers, and granulocytes in the blood [39]. Research suggests that EXFABP may play a significant role in regulating hen immunity and egg production rates [40]. Furthermore, EXFABP is closely associated with the differentiation of chicken fetal myoblasts, muscle growth, and energy metabolism [41]. To date, there is a lack of studies on the mechanisms by which EXFABP affects egg production in hens, warranting further investigation.

In our subsequent analysis of the cis-regulatory target genes of SGK1-OT1 and LINC2698, in addition to the cis-regulation of SGK1 by SGK1-OT1, we identified three genes: SLC2A12, TBPL1, and C2H8ORF22.

SLC2A12 encodes a protein belonging to the glucose transporter (GLUT) family, which plays a crucial role in the intracellular transport of glucose, thereby participating in numerous physiological processes. It has been reported that SLC2A12 regulates glucose utilization in chicken skeletal muscle, with its expression increasing during embryogenesis and post-hatching, supporting its role in ECM remodeling and glucose metabolism during follicle maturation and ovulation periods [42]. Additionally, histomorphological examinations of the ovaries of female rabbits stimulated with gonadotrophins, coupled with whole-genome miRNA and mRNA transcriptome analyses, have identified SLC2A12 as a promising downstream target of pregnant mare serum gonadotrophin (PMSG) and human chorionic gonadotrophin (hCG) [43]. Efficient egg production efficiency encompasses the number of follicles, as well as the ovaries' capacity for ovulation and transforming follicles into hard-shelled eggs [44]. Given that the protein encoded by the SLC2A12 gene is involved in the intracellular transport of glucose, it is conceivable that it may supply energy to ovarian follicles, thereby impacting the egg-laying capacity of chickens. Further research is required to confirm this hypothesis. TBP (TATA-box Binding Protein) is a critical factor required for the initiation of transcription in eukaryotes, serving as a key molecule in gene regulation through its interactions with transcription regulatory factors and the basic transcription machinery. TBPL1 (also known as TRF2) represents a paralog of TBP in metazoans, playing a significant role in zygotic transcription during the embryogenesis of organisms such as Caenorhabditis elegans and Drosophila melanogaster [45–47]. Research has indicated that TBP variants, such as TBPL1 and TBPL2, may regulate developmental processes including gametogenesis and differentiation, myogenesis, and ventral patterning during development [48]. The study of TBP and its associated factors is of paramount importance for elucidating the molecular mechanisms underlying gene regulation. Research on the C2H8ORF22 gene is relatively scarce, with mentions in transcriptomic analyses of avian heart and liver tissues [49].

We found that numerous lncRNAs target genes and mRNAs were both involved in the regulation of neuroactive ligand-receptor interaction, CCR6 chemokine receptor binding, G-protein coupled receptor binding, Cytokine-cytokine receptor interaction and ECM-receptor interaction. Neuroactive ligands affect neuronal function by binding to intracellular receptors, which have the capability of binding transcription factors and regulating gene expressions [50]. Mu et al.’s study suggested neuroactive ligand-receptor interaction pathway might affect egg production in chickens via a mechanism similar to that found in fish [51]. Caballero-Campo et al. demonstrated that CCR6 protein is localized on the surface of human sperm [52]. Chemokines play various biological functions by activating surface receptors of their target cells, and also have the ability to interfere with sperm–oocyte interaction [53]. In addition, CCR6 specifically binds to its ligand chemokine CCL20. Duan et al. demonstrated that the chemokine CCL20 is abundantly present in human follicular fluid and is produced by human oocytes as well as surrounding cumulus granulosa cells [54]. This chemokine has an important function for the process of fertilization. G protein-coupled receptors (GPCRs), representing the largest protein family encoded by the human genome, are membrane-bound receptors that mediate crucial physiological responses by converting extracellular signals. These receptors exhibit a diverse array of endogenous ligands, encompassing odorants, hormones, neurotransmitters, and chemotactic factors. The ligands for GPCRs span a broad spectrum, ranging from photons, amines, carbohydrates, lipids, peptides, to proteins [55]. In the ovary, multiple GPCRs play a role in regulating reproductive functions. These receptors interact with endogenous hormones, neurotransmitters, or other signaling molecules within the ovary, thereby influencing important physiological processes such as ovarian development, follicle growth, and ovulation. Investigation of these GPCR signaling pathways contributes to a better understanding of the regulatory mechanisms underlying ovarian function. Cytokines are soluble extracellular proteins or glycoproteins that play critical roles as intercellular mediators and mobilizers in innate and adaptive immune host defense, cell growth, differentiation, cell death, angiogenesis, and developmental and reparative processes aimed at restoring homeostasis. They are released by various cells in response to activating stimuli and induce specific biological responses by binding to specific receptors on the cell surface of target cells. Existing research has provided evidence supporting the significant involvement of the cytokine-cytokine receptor interaction pathway in follicle development [56]. The extracellular matrix (ECM) is a complex matrix of biomacromolecules, including glycoproteins, proteoglycans, and glycosaminoglycans. The ECM-receptor interaction pathway is the most significantly enriched signaling pathway in terms of gene enrichment. It plays a pivotal role in various aspects of cellular physiological activities, such as cell adhesion, migration, proliferation, and differentiation [57]. Transcripts associated with the "ECM-receptor interaction" pathway may complement the enrichment of Gene Ontology categories related to cell adhesion in biological processes, indicating their potential significance in promoting cell adhesion and cohesion. This evidence underscores the crucial molecular-level importance of adhesion and cohesion mechanisms in the physiological activities of the ovary [58].

## Conclusion

In this investigation, we executed transcriptomic sequencing on ovarian tissues from BC and GC groups within TBsf, identifying 349 significantly differentially expressed mRNAs and 651 lncRNAs. Notably, two pivotal lncRNAs—SGK1-OT1 and LINC2698—along with their target genes STC1, IL8, C6orf58, and EXFABP, were discerned as potential regulators impacting chicken reproductive performance. Via extensive GO and KEGG analyses, we delineated five pathways integral to egg production: neuroactive ligand-receptor interaction, CCR6 chemokine receptor binding, G-protein coupled receptor binding, cytokine-cytokine receptor interaction, and ECM-receptor interaction. The elucidation of these genes and pathways provides critical insights into the mechanisms underlying broodiness behavior. Consequently, our research lays the groundwork for identifying functional genes and validating key traits in TBsf, offering strategic directions for future molecular breeding programs aimed at enhancing egg-laying efficiency.

## Materials and methods

### Animal and sample collection

Eight 30-week-old female TBsf chickens, including four exhibiting broodiness (BC) and four with high egg-laying rates (GC), were acquired from the Taihe Aoxin Black-Bone Silky Fowl Development Co., located in Taihe County, Jiangxi Province, China.

To categorize the TBsf based on their brooding behavior and egg-laying capacity, we conducted a continuous collection and recording of egg-laying patterns. The chickens were reared under identical housing and feeding conditions. In our study, euthanasia was carried out in strict accordance with the guidelines recommended by "the American Veterinary Medical Association (AVMA) Guidelines on Euthanasia". Our primary concern was to ensure the process was humane and minimized any distress or pain to the animals involved. To this end, animals were first anesthetized using Isoflurane, followed by cervical dislocation. The choice of Isoflurane was based on its rapid action and minimal discomfort to the animals. The animals were administered a 4% concentration for induction and 2% for maintenance, using an inhalation chamber, to ensure they were unconscious and free from pain during the euthanasia procedure. Following humane euthanasia and necropsy, ovarian tissues were harvested, immediately flash-frozen in liquid nitrogen, and preserved at − 80 °C for subsequent analysis.

### RNA isolation, library construction and sequencing

Total RNA was extracted from each sample using TRIzol reagent (Invitrogen, provided by Shanghai Solarbio Science & Technology Co., Ltd., Shanghai, China). The integrity of the RNA and the presence of any DNA contamination were verified through agarose gel electrophoresis. NanoDrop spectrophotometry was used to determine the purity and concentration of the total RNA. RNA integrity was further confirmed with an Agilent 2100 Bioanalyzer. Samples with A260/A280 ratios between 1.8 and 2.0 and RNA Integrity Number (RIN) values equal to or greater than 7.0 were considered to be of acceptable quality. Ribosomal RNA (rRNA) was then depleted from the total RNA, which was subsequently fragmented into short segments of 250 to 300 base pairs (bp). First-strand cDNA synthesis was initiated using these RNA fragments as templates and random oligonucleotides as primers, followed by the synthesis of the second cDNA strand using a mix of dNTPs, including dUTP. The resulting double-stranded cDNA underwent end-repair, A-tailing, and adapter ligation. cDNA fragments around 350–400 bp were selectively purified using AMPure XP beads. The cDNA containing uracil in the second strand was degraded with the USER enzyme (New England Biolabs, Massachusetts, USA). After PCR amplification, eight libraries were constructed. These libraries were sequenced on an Illumina NovaSeq 6000 platform, using a 150 bp paired-end (PE150) protocol by Novogene Corporation (Beijing, China).

### Bioinformatics analysis

Initially, raw sequence reads in FASTQ format were processed using a custom Perl script to ensure data quality and reliability. This step involved the removal of low-quality reads that contained adapters, poly-N sequences, and bases below quality threshold, followed by the calculation of Phred quality scores (Q20 and Q30) and the GC content to obtain clean reads. All downstream analyses were performed on these high-quality data sets. Paired-end clean reads were then aligned to the reference genomes (available at Ensembl: release-105 for Gallus gallus) using Hisat2 to assemble the most comprehensive transcriptome possible [59]. StringTie was employed to splice and quantify these aligned reads into transcripts [60]. We utilized Cuffmerge to integrate transcripts from various samples, discarding any with ambiguous chain direction or lengths shorter than 200 nucleotides. The expression levels of transcripts were quantified using FPKM values, as calculated by StringTie. Subsequently, edgeR was applied to identify differentially expressed lncRNAs and mRNAs, with statistical significance assigned to those showing differential expression.

### Co-expression (trans) and co-location (cis) analyses

Cis-acting lncRNAs influence proximal protein-coding genes; thus, we considered protein-coding genes located within a 100-kb region up- or downstream of an lncRNA as cis-regulated targets. For trans-regulated target genes, we assessed the correlation between the abundance of identified lncRNAs and the expression of known protein-coding genes in both laying and brooding chickens. We specifically selected those with a Pearson correlation coefficient (|r|) greater than 0.95 for further examination. Furthermore, we employed Cytoscape v3.10.0 to visualize networks of differentially abundant lncRNAs and their associated cis- and trans-regulated target genes, delineating the intricate lncRNA-gene interactions.

### GO and KEGG enrichment analysis

To gain deeper understanding of the functions and classifications of the differentially expressed genes and the target genes of lncRNAs, we carried out Gene Ontology (GO) term and Kyoto Encyclopedia of Genes and Genomes (KEGG) pathway analyses using the GOseq and KOBAS software. The GO database is an extensive resource that categorizes gene functions into three domains: molecular function, biological process, and cellular component. The KEGG database offers a systematic compilation of genomic, chemical, and functional information [61]. We considered a q-value of less than 0.05 as indicative of significant enrichment among the differentially expressed genes.

### qRT-PCR Validation

For real-time quantitative PCR (qRT-PCR) analysis, we collected four samples from each of the two groups. Each qRT-PCR procedure was performed in triplicate to ensure accuracy. We selected three lncRNAs and six mRNAs for detection via qRT-PCR. β-actin served as the reference gene. We calculated the relative expression levels of the genes and lncRNAs using the 2^−∆∆Ct^ method. Details of the primer sequences used for the lncRNAs and mRNAs can be found in Table S12.

### Supplementary Information

**Supplementary Material 1.**

**Supplementary Material 2.**

**Supplementary Material 3.**

**Supplementary Material 4.**

**Supplementary Material 5.**

**Supplementary Material 6.**

**Supplementary Material 7.**

**Supplementary Material 8.**

**Supplementary Material 9.**

**Supplementary Material 10.**

**Supplementary Material 11.**

**Supplementary Material 12.**

## Data Availability

All data generated or analyzed during this study are included in this published article and its additional files, or in the following public repositories. Data have been submitted to a public database under the accession number PRJNA1006164 (https://www.ncbi.nlm.nih.gov/bioproject/?term=PRJNA1006164).

## Abbreviations

lncRNAs: Long non-coding RNAs
BC: Broody Chicken
GC: High egg-laying chicken
TBsf: Taihe Black-Bone Silky Fowls
DE: Different Expression
GO: Gene Ontology
KEGG: Kyoto Encyclopedia of Genes and Genomes
DEGs: Differentially Expressed Genes
FPKM: Fragments per kilobase of transcript per million mapped reads
BP: Biological processes
CC: Cellular components
MF: Molecular functions
HPG: Hypothalamic-pituitary–gonadal
pFF: Porcine follicular fluid
ORFs: Open reading frames
PMSG: Pregnant mare serum gonadotrophin
hCG: Human chorionic gonadotrophin
qRT-PCR: Quantitative real-time PCR
